# Ogilvie’s Syndrome Secondary to Narcotics Complicated by Colonic Perforation

**DOI:** 10.7759/cureus.45173

**Published:** 2023-09-13

**Authors:** Lauren Punter, Raydiene Doorgen, Penelope Mashburn

**Affiliations:** 1 Surgery, American University of Antigua, St. John's, ATG; 2 Surgery, Trumbull Regional Medical Center, Warren, USA

**Keywords:** general surgery, opioids, narcotics, pseudo-obstruction, colonic perforation, ogilvie's syndrome, colonic pseudo-obstruction

## Abstract

Ogilvie's syndrome is a condition characterized by colonic dilation in the absence of mechanical obstruction. This case report presents a patient who was immobile and hospitalized for several months following a motor vehicle accident that left the patient paralyzed, who subsequently developed Ogilvie's syndrome. The aim of this study is to briefly discuss the clinical presentation, diagnostic workup, and management of Ogilvie's syndrome. This article also discusses the impact of narcotics and the possible association with Ogilvie's syndrome in this patient.

## Introduction

Ogilvie's syndrome, also known as acute colonic pseudo-obstruction, is characterized by colonic dilation that is not due to an identifiable mechanical or anatomical cause but is more likely due to a severe motility disturbance. It is common in elderly patients with multiple comorbidities and immobile patients in association with a severe illness, and it can be secondary to medications such as narcotics, anticholinergics, and anticonvulsants and may also occur as a complication following surgery [1-3].

The diagnosis of Ogilvie's syndrome hinges on exclusion. Signs and symptoms include worsening abdominal distention and pain and sometimes accompanied by fever, nausea, and vomiting. These typically present over three to five days but can occasionally present acutely within 48 hours. Diagnostic confirmation often involves imaging techniques like upright and supine abdominal X-rays. Management strategies predominantly emphasize conservative measures, including addressing underlying causes, correcting electrolyte imbalances, minimizing or discontinuing potential inducing medications, bowel rest, and decompression using nasogastric or rectal tubes [1].

## Case presentation

Our patient, a 43-year-old male, suffered paraplegia following a motor vehicle accident approximately eight months ago. His medical history is marked by myocardial infarctions and severe protein-calorie malnutrition. The patient had a prior percutaneous endoscopic gastrostomy (PEG) placement and was receiving tube feeds daily. Due to incontinence, he also has a colostomy for fecal incontinence and a suprapubic catheter for urinary incontinence. He presented to the emergency department complaining of a cessation of ostomy output over the past four days, despite ongoing oral intake. Additionally, he was taking narcotics for chronic back pain. General surgery was consulted to evaluate the diminished ostomy output. On physical examination, his abdomen was soft, non-tender, and moderately distended, with no signs of rebound tenderness or guarding. The ostomy appeared viable in the left lower quadrant of the abdomen, with minimal serosanguineous output.

**Figure 1:**
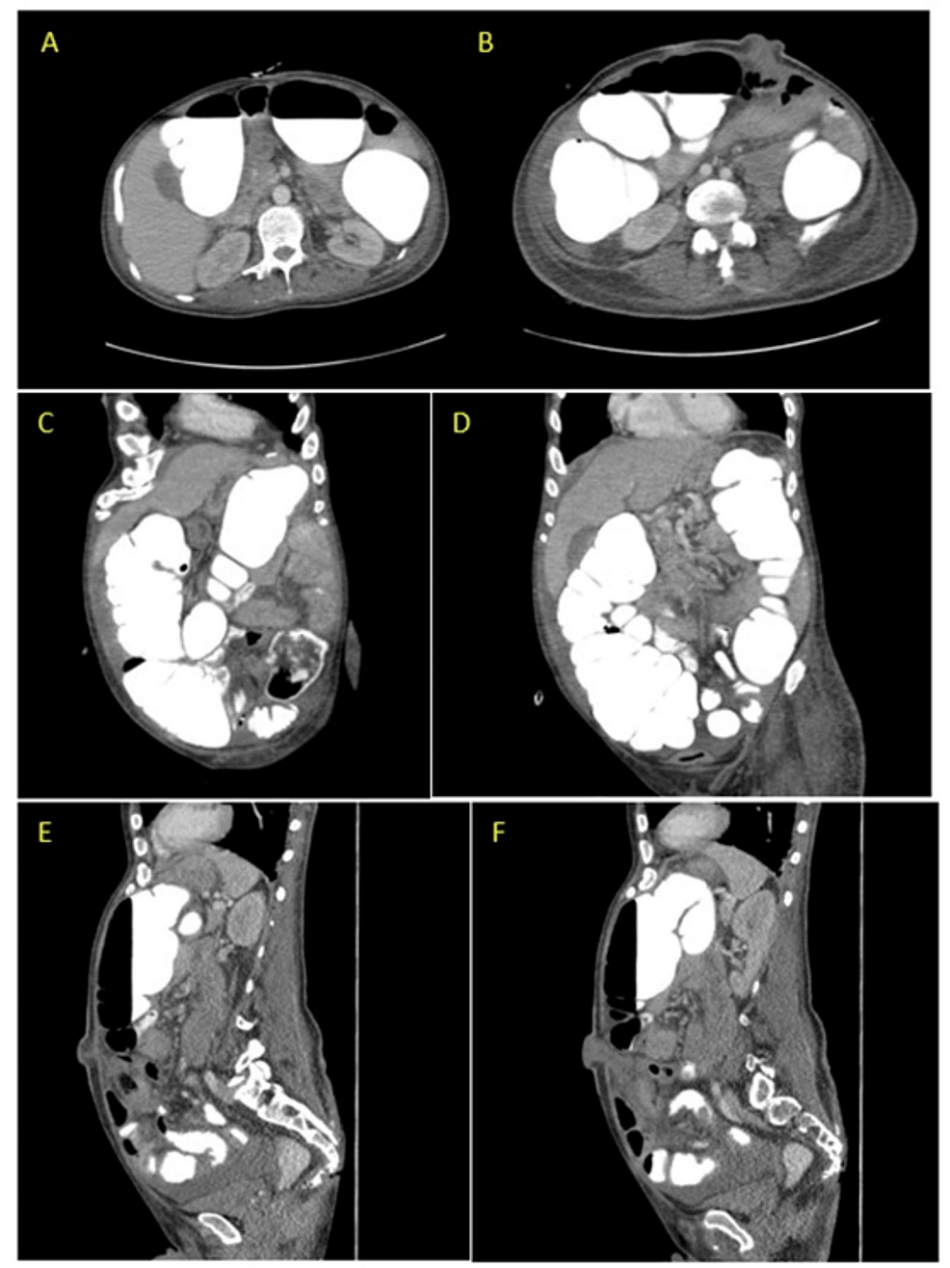
CT abdomen and pelvis with oral and IV contrast showing marked distention and colonic dilation without anatomic obstruction. Images A and B are the axial view and C, D, E, and F are the sagittal view, with E and F showing the ostomy

A contrast-enhanced CT scan of the abdomen and pelvis revealed normal small bowel loops and marked colonic distention with contrast throughout the colon (Figure 1). However, the patient's ostomy output remained diminished, suggesting ileus. Consequently, the patient's narcotic medications were discontinued, and he was placed on nil per os (NPO) status, with the PEG tube set to gravity for conservative management. The following day, the ostomy was digitally examined and found to be patent without stenosis or obstruction. Subsequently, the patient underwent a colonoscopy to further evaluate the ostomy site, revealing minimal fecal matter but no obstruction. A diagnosis of colonic pseudo-obstruction was established.

The patient was given soap suds enemas, and this caused the ostomy output to be increased over the following two days. The patient’s condition was continuously monitored and eventually, he transitioned to a clear liquid diet. However, two days later, he developed tachypnea, hypotension, and tachycardia, indicative of sepsis, with an elevated lactic acid level of 3.3 mmol/L. He was promptly transferred to the intensive care unit for stabilization and close monitoring overnight. A kidney, ureter, and bladder X-ray (KUB) conducted that night showed a mild decrease in colonic distention without evidence of pneumoperitoneum.

**Figure 2:**
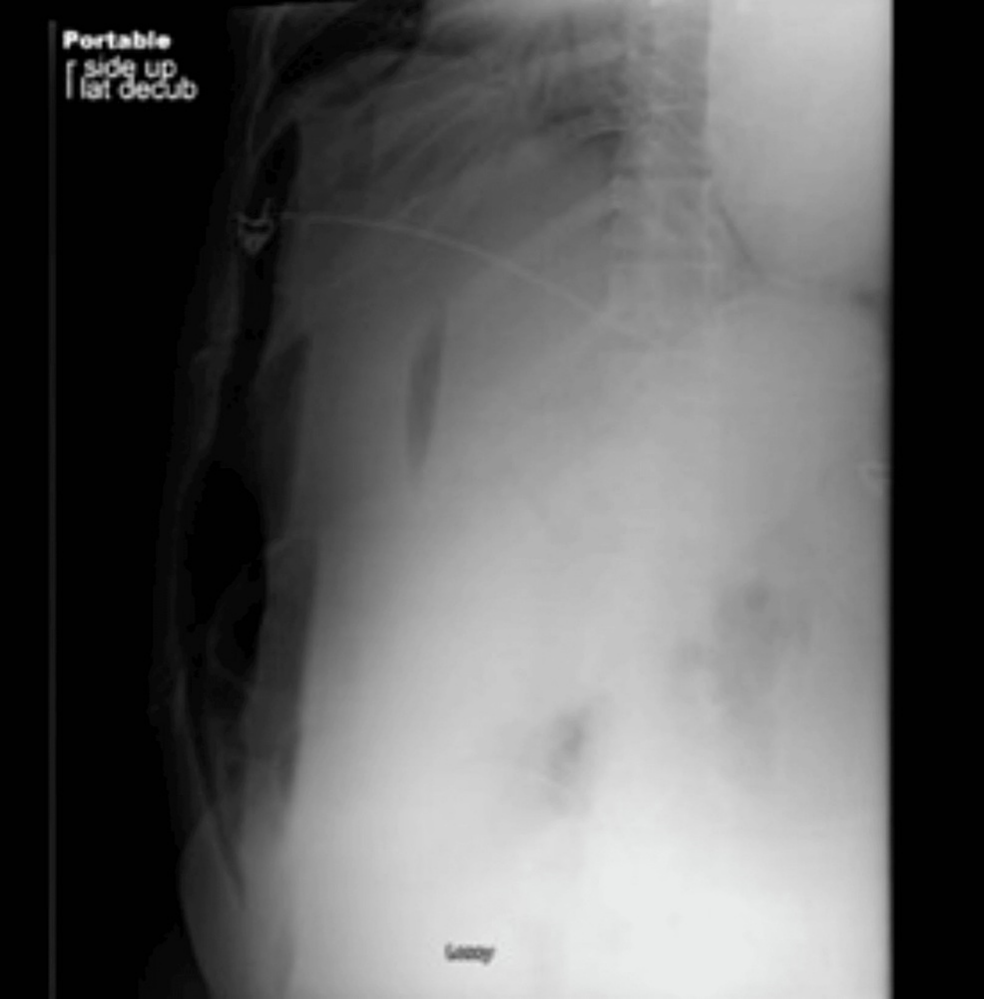
KUB in the left lateral decubitus that showed significant pneumoperitoneum

The following morning, the patient experienced a cardiac arrest, undergoing cardiopulmonary resuscitation for about two minutes, and the patient thereafter achieved a return of spontaneous circulation without the need for epinephrine. A repeat KUB in the left lateral decubitus then showed significant pneumoperitoneum, prompting the patient to be taken to the operating room (Figure 2).

The end colostomy was sutured closed, and upon entry into the abdomen, feculent peritonitis and a substantial amount of free fluid and succus throughout the abdominal wall cavity were noted. The entire remaining colon was noted to be grossly distended and ischemic, with perforation in the sigmoid colon just below the descending colostomy. There were no signs of perforation or ischemia noted in the small bowel. The PEG tube was found to be intact within the stomach lumen. Given the extensive colonic distention and ischemia, the decision was made to proceed with a subtotal colectomy.

The colostomy was taken down to the level fascia and returned to the abdomen. The descending colon was then mobilized laterally to medially, and the dissection was made distally along the colon to the splenic flexure. A mesenteric window was created proximal to the terminal ileum, where the bowel appeared healthy and well-perfused. Subsequently, the terminal ileum was transected, and the abdominal cavity was irrigated with saline until clear fluid return was achieved. The ileum was brought through, and an end ileostomy was created at the previous colostomy site, as there were no gross signs of infection and appropriate sizing.

The abdominal fascia was closed, but due to the extent of contamination from feculent peritonitis, the midline incision was intentionally left open. The midline wound was packed, and three days after the exploratory laparotomy involving a total abdominal colectomy and an end ileostomy, the patient underwent delayed primary closure of the abdominal wall incision using staples at the bedside.

Given the amount of feculent peritonitis contamination, a decision was made to leave the midline incision open. The midline wound was packed, and three days after the exploratory laparotomy which included total abdominal colectomy and end ileostomy, the patient underwent delayed primary closure of the abdominal wall incision using staples at the bedside.

## Discussion

Pseudo-obstruction occurs when the propulsive forces of intestinal peristalsis become incapable of overcoming natural resistance to flow, resulting in a clinical presentation that resembles mechanical bowel obstruction [4]. X-ray imaging is used to measure the degree of initial colonic dilatation and for serial imaging during observation after diagnosis is made. CT scan with oral and IV contrast is the preferred modality for diagnosis. The preferred diagnostic modality is a CT scan with oral and IV contrast, revealing bowel wall thickening and colonic dilation without anatomical obstruction [1].

Patients with chronic pain often rely on narcotics for symptom management. Opioids, including fentanyl, oxycodone, and hydromorphone, are cornerstone treatments for moderate to severe acute and chronic pain, particularly in palliative care. Common side effects encompass drowsiness, confusion, hallucinations, nausea, vomiting, and constipation [5,6]. Notably, opioids can contribute to the development of Ogilvie's syndrome, potentially through dysregulation of neurotransmitters like acetylcholine, nitric oxide, and vasoactive intestinal peptide, affecting the autonomic nervous system. These medications can significantly disrupt bowel motor activity, making the cessation of the offending drug the primary treatment for opioid-induced enteric dysmotility [4].

Endoscopic decompression, performed via colonoscopy with minimal air insufflation, serves both diagnostic and therapeutic purposes. If there is no improvement within 48 hours, neostigmine, an acetylcholinesterase inhibitor, may be administered. Neostigmine exerts its therapeutic effect on pseudo-obstruction by stimulating both nicotinic and muscarinic receptors, enhancing parasympathetic activity and resulting in hyperperistalsis. Intravenous neostigmine is administered at doses ranging from 2 to 5 mg over 3 to 30 minutes in patients with Ogilvie's syndrome [4,7].

Some patients may fail to respond to initial management and face having a prolonged recovery period which may lead to an increased risk of complications. The greater the colonic dilation, the greater the wall tension which can lead to spontaneous perforation, bowel ischemia, or peritonitis [2]. While the presence of free air on acute abdominal series raises concerns for perforation, it does not conclusively confirm it. When there is perforation or gangrene of the bowel, surgery should be performed and the affected region resected [7].

In this case, the patient's transition from a previously healthy state to becoming paraplegic and immobile for several months, coupled with the use of medications like morphine, could have substantially contributed to the development of Ogilvie's syndrome. Despite the emergence of complications that led to perforation and subsequent feculent peritonitis, effective management strategies were employed, ultimately leading to successful treatment. As a result, the patient was discharged to a long-term acute care facility while maintaining a functioning ileostomy and remaining in a stable condition.

## Conclusions

Given the limited knowledge and scarcity of studies regarding the development of Ogilvie's syndrome secondary to opioids, we have chosen to share our experience with this case through publication. Prompt discontinuation of medications such as opioids and anticholinergics is essential. Daily laboratory tests and abdominal imaging played a pivotal role in monitoring colonic dilation progression. Management of Ogilvie's syndrome involves initiating an NPO regimen and employing a nasogastric tube for decompression. Notably, decompression colonoscopy has demonstrated both safety and effectiveness. Surgical intervention becomes necessary if conservative management proves ineffective or if concerns arise regarding ischemia, necrosis, or perforation, as observed in this patient's case. In conjunction with discontinuing the patient's medication and initially pursuing conservative measures, the patient's deteriorating condition, culminating in perforation, necessitated surgical intervention, ultimately culminating in a subtotal colectomy as the most viable option.

This case underscores the critical importance of recognizing and managing Ogilvie's syndrome, particularly in patients with risk factors like opioid medication usage and immobility. It also highlights the need for further research and exploration into the relationship between opioids and the development of this condition.
